# An Exploratory Study of Consumers’ Perceptions of Product Types and Factors Affecting Purchase Intentions in the Subscription Economy: 99 Subscription Business Cases

**DOI:** 10.3390/bs12060179

**Published:** 2022-06-06

**Authors:** Hyehyeon Baek, Kilsun Kim

**Affiliations:** College of Business, Sogang University, Seoul 04107, Korea; phhgood8282@gmail.com

**Keywords:** subscription model, utilitarian goods, hedonic goods, search goods, experience goods, context effects

## Abstract

This study examined changes in consumer perceptions of product types and purchase intentions when a subscription model is introduced for products normally sold on a one-time basis. It then proposed product types likely to affect consumers’ purchasing intentions in the subscription economy and product categories best suited for the subscription economy. To this end, an experimental study was conducted with experts and general consumers using 99 subscription business cases. It was found that a regular delivery of products on a subscription basis gradually changes consumer perceptions of the products from utilitarian to hedonic and from search to experience ones. It was also found that consumption motivation is an important predictor of consumer purchase intentions in the subscription economy. In addition, experience-utilitarian and search-utilitarian products were associated with the highest purchase intentions among experts and general consumers, respectively. This suggests that a company’s strategy should be adjusted in line with consumers’ understanding of the subscription model. Therefore, suppliers need to understand the full implications of the new model, such as changed consumer perceptions and purchasing intentions, and strive to design a subscription model that is suitable for the target segments and product selections.

## 1. Introduction

The past decade has witnessed a rapid growth of subscription-based business models where customers pay a fixed fee at regular intervals to receive access to a product or service [1,2,3]. A subscription business model presupposes a long-term provider–customer relationship [4] based on a periodic payment (e.g., a monthly fee) which entitles the customer to unlimited use of the contents until the customer cancels the subscription. Various subscription models have recorded a tremendous growth, marking a 30-fold increase in demand between 2013 and 2016 alone [5]. The proliferation of the subscription model can be primarily attributed to the online distribution of digital content (e.g., Netflix and Spotify), and this new business model has been adopted by a growing number of businesses across a wide range of industries, including food, clothing, software, automobiles, airline tickets, and healthcare services. The global outbreak of COVID-19 has further accelerated the spread of subscription-based businesses across the globe [6]. According to a recent industry report, subscription-based businesses have grown by an enormous 437% in the last decade [7].

Subscription-based business models can be seen as a new business model innovation [8], as a game changer or breakthrough which can reshape the traditional market models and drastically influence consumers’ behavior, even to the extent of being seen as a potential disruptive innovation [9,10,11]. As verified in several literature reviews, customer perception of a new business model is a viable predictor of customer success [12,13,14]. 

However, despite a significant increase in subscription-based businesses, very little is known about the factors that attract consumers to subscription offers or the barriers to commitment [15]. To be successful in leveraging these new business models, companies have to be able to understand how to create value for the users and how to avert the pitfalls that might deter their potential customers from subscribing [8]. Consumer perception is an important area of discussion when choosing a right way to market a business model [16,17] but it has not yet been sufficiently studied in relation to subscription models. There is limited knowledge of whether consumers’ behavior differs across different types of subscription services [15,18,19]. 

Just as consumers’ emotional involvement varies depending on product type [20], their information processing and decision-making activities may also vary depending on product type [21,22,23]. Therefore, it is essential for suppliers to closely look at the characteristics of the products they offer and incorporate consumer-perceived product classifications into their analysis [24].

It is necessary to investigate whether there is a change in consumers’ perceptions of product type and their purchase intentions when the subscription model is applied. According to behavioral decision research, when placed in a specific contextual choice situation, consumers tend to apply new choice rules adjusted to the given context instead of the existing ones [25,26]. A subscription model changes delivery channels of the products that used to be sold on a one-time basis. This allows us to infer that the subscription model may create effects that induce consumers to perceive the existing products differently, stimulated by the changed context in which the products are offed, thus supporting the context effect theory. 

In this context, this study investigated consumers’ perceptions of product types and purchase intentions for 99 products offered on a subscription basis. In so doing, it was also intended to identify the main variables influencing purchase intentions and the product type categories most suitable to be offered as a subscription service based on consumers’ perceptions. The results of this study are expected to help suppliers select suitable products for subscription models and to set up and implement optimal sales strategies accordingly, thus contributing to expanding the scope of research on the subscription economy.

The remainder of this paper is organized as follows. Section 2 presents the hypotheses based on the theoretical background. Section 3 introduces the measurement concept and the research methodology, while Section 4 describes the analysis process and presents the results based on the research questions. Section 5 discusses the results, and Section 6 describes the implications and limitations of the study results and provides suggestions for future research.

## 2. Background and Hypotheses

### 2.1. Subscription Models

Subscription models are designed to solve individual customers’ problems and meet their changing requirements [27]. Today’s consumers go beyond purchasing what the company has made and want the company to take a closer look at what the consumer wants, and choose the company’s product or service that satisfies that need as much as possible. It is the subscription economy that has emerged along with these changes in consumption [28]. This includes constantly providing products and services updated according to the customers’ changing requirements, which inevitably results in a constant change of the business model [27]. This aspect has been examined in a small number of recent studies examining the factors influencing consumers’ motivation to use subscription models and the purchase intentions that providers should consider [29,30,31,32]. 

For example, as Woo and Ramkumar [33] explained, the convenience and stimulation of the subscription-based online services (SOS) shopping matches the needs of the modern consumers in terms of time-saving and hedonic shopping experience. Similarly, Chiu et al. [34] investigated the utilitarian and hedonic variables in explaining purchasing behavior in e-commerce environments, which also constitute viable platforms for subscription models. Their analysis revealed that the risks perceived in e-commerce environments undermine repurchase intentions and moderate the effects of utilitarian and hedonic values on repurchase intentions. The findings of these studies indicate that consumers’ utilitarian and hedonic values can affect their attitudes and behavioral intentions of repeat purchase and that utilitarian value has a greater influence on the related factors than does hedonic value [30,33,34]. Additionally, Jeong [35] identified utilitarian and hedonic motivations as well as and consumer innovativeness as significant variables influencing consumer attitudes and intention to utilize subscription services. Furthermore, consumer attitudes toward subscription-based online services (SOS) were observed to mediate the relationship between utilitarian motivation, hedonic motivation, consumer innovativeness, and use intention. Lee et al. [36] performed a quantitative study to identify the main stimuli (product price, quality, assortment, uniqueness, and surprise) that influence consumer loyalty and found that product quality and uniqueness affected consumer attitudes toward products in the subscription economy. Finally, Bray et al. [15] explained that given different characteristics, consumer attitudes and behaviors can vary depending on the type of subscription service under consideration. They highlighted the importance of conducting more research to better understand these consumer behaviors for suppliers to target their customers more efficiently. This study highlights the utilitarian motives such as convenience and ease for purchase have greater influence on subscription models.

This study differentiates itself from previous research on subscription models in that it first examined possible changes in consumers’ perceptions of product types and their purchase intentions when existing products are offered under a subscription model and further explored which product categories lend themselves better to the subscription economy than others.

### 2.2. Context Effects

The term “context effect” refers to the altered perception of a stimulus caused by the formation of a new context around it and is mainly discussed in cognitive psychology [37]. From a marketing perspective, context effects have been viewed as a major determinant of consumers’ product choice behavior [38]. On a related note, context effects have been studied from various aspects such as product attributes [39], selection constraints [40], and external constraints [41]. Research in the field of business administration pays a great deal of attention to the phenomenon that consumer preferences tend to change in situations where a product provides options among various conflicting properties [42,43]. Wade et al. [44] reported that providing the same content in different formats had varying effects on participants’ interest and performance. In this respect, Kwon et al. [45] suggested that the effectiveness of advertisements appealing to emotions or moods is mainly governed by the context effects arising from the emotional tone of media. In a meta-analysis on the relationship between media context and attitudinal outcome measures, they noted that various facets of media context make differentiated use of consumers’ attitudes and purchase intentions. Kahn and Sarin [46] used context effects to present a model designed to predict consumer choices in uncertain and ambiguous situations. Specifically, they emphasized the importance of exploring contextual trait relationships that influence consumer attitudes to better understand contest effects. 

To date, no research has been dedicated to examining the change in consumer perceptions of the same product triggered by the provider’s adoption of the subscription model which is offered to the already existing consumers as a new purchase context. We hypothesize that the subscription model essentially creates a new purchase context that promotes more long-term regular consumer–provider relationships in an interactive environment, with added possibilities of personalization and customization to meet changing consumer needs and requirements. Therefore, based on the framework of context effect theory, the subscription model can produce effects that differentially stimulate and change consumers’ perceptions of existing products. The subscription model changes the way how existing products are delivered, rather than providing new products.

### 2.3. Product Types

In a literature review it was found that product types can be categorized into utilitarian and hedonic goods based on consumption motivation, and search and experience goods based on quality inference.

#### 2.3.1. Consumption Motivation: UTILITARIAN versus Hedonic Goods

In terms of consumption motivation, products are classified into two major categories: utilitarian goods and hedonic goods [23,47,48,49]. Consumers buy utilitarian goods for practical purposes and hedonic goods to increase their happiness [50]. Utilitarian goods possess practical attributes that serve functional roles, whereas hedonic goods possess pleasure-seeking attributes, including sensory pleasures [20,47,51]. Therefore, functionality is an important factor for products that consumers perceive as utilitarian goods, while consumers attach more importance to the joy, satisfaction, and delight derived from products they perceive as hedonic goods [52].

Utilitarian and hedonic goods are not mutually exclusive; some products may possess both utilitarian and hedonic attributes to a lesser or greater extent. However, even if a product possesses mixed properties, consumers may categorize it as either a utilitarian or a hedonic product depending on whether they perceive it to be more fundamentally utilitarian or hedonic [20]. This suggests that consumers may purchase the same products for varying reasons.

From a supplier perspective, it is crucial to understand whether consumers perceive their products as utilitarian or hedonic goods since consumption motivation influences the amount of money and time (effort) that consumers are willing to invest when making purchase decisions [53]. Specifically, prior studies have revealed that consumers are more likely to experience negative emotions when choosing hedonic goods compared with utilitarian goods [21,54]. Researchers have also found that consumers spend more money on utilitarian goods than on hedonic goods, making purchase decisions more easily when buying utilitarian products [21,54]. For these reasons, companies should understand whether consumers perceive utilitarian or hedonic values when purchasing their products and set up their marketing strategies accordingly.

#### 2.3.2. Quality Inference: Experience versus Search Goods

Product types can also be categorized into search goods and experience goods depending on whether consumers can evaluate their quality prior to purchase or only after purchasing or experiencing them [55,56,57]. Search goods have attributes that can be evaluated prior to purchase based on externally available information, while experience goods have attributes that can be evaluated only through direct personal observation or use. Hence, the quality of search goods can be assessed based on the technical information such as product specifications, whereas that of experience goods can be assessed only through direct and tangible interaction [24]. Search and experience goods can typically be distinguished by the product satisfaction experienced by consumers in the purchase process [57]. Consumers are dissatisfied when product quality information acquired prior to purchase deviates from the actual quality evaluated after purchase [58,59]. Moreover, searching for information itself can be a source of enjoyment for many customers [60]. A practical implication of these findings is that providers should choose differentiated merchandising channels and communication strategies based on their consumers’ perceived quality inferences.

Chiang and Dholakia [24] investigated whether consumers’ online purchase intentions vary depending on product type and found that online purchase intentions were greater for search goods than for experience goods. In a similar vein, Wright and Lynch [59] reported that advertising through media (e.g., TV or newspapers) is more effective than direct experience for products with strong search attributes, and the other way around for products with strong experience attributes. Accordingly, they suggested that it would be more effective for stores to appeal to experience attributes and for distribution channels, such as home shopping and catalog shopping, to appeal to search attributes. 

Meanwhile, Peterson et al. [61] argued that the traditional dichotomy of search versus experience attributes should be reviewed in the light of the new opportunities provided by the Internet and highlighted the importance of integrating product or service attributes into the discussion surrounding the use of the Internet as a marketing tool. Similarly, Klein [55] explained that the Internet or the new interactive media undermines the meaning assigned to traditional experience vs search goods. Since consumers can acquire information on experience goods through online platforms even prior to purchase without directly experiencing them, the experience attributes inherent in these products undergo dynamical changes in quality inference. Preliminary experiments by Huang et al. [62] showed that there are significant differences in consumers’ cognitive ability to evaluate pre-purchase product quality between search and experiential goods in a traditional retail environment, but these differences are blurred in an online environment.

### 2.4. Hypotheses

The research hypotheses were formulated based on the research questions of this study as follows:

Research on behavioral decision-making that deals with consumer preferences or alternative choices argues that, when a consumer is placed in a specific situation, he/she tends to make decisions based on the given context rather than on the previously established choice rules [25,26]. In other words, when faced with a specific purchasing situation, a consumer’s purchase decision generates a new preference based on the context of the given situation rather than existing product preferences. Several studies on context effects support this argument [42,43,63]. If so, will the subscription model is introduced for an existing product, will consumer perceptions of the product’s type and their purchase intentions change? (RQ 1).

A subscription model may have a context effect that can sufficiently stimulate customers’ perceptions and affect their purchase behavior such that their experiences with existing products and services are converted into lasting relationships rather than one-time sales. Therefore, we hypothesized that, if the supply method of an existing product is changed under a subscription model, change in consumer perceptions will also occur through context effects. Accordingly, RQ1 investigates the influence of the subscription economy by exploring whether consumers’ perceived consumption motivation and quality inference, in addition to purchase intentions, change when a subscription model is introduced.

Specifically, previous research has demonstrated that the subscription model provides customers with pleasure motivations such as surprise and excitement [29]. It has also been reported that the convenience and new stimulation provided by the subscription model perfectly fit the modern consumer’s hedonistic shopping experience [64]. Therefore, this study argued that when a subscription model is applied to an existing product, consumption motivation will tend to change from utilitarian to hedonic motives.

**H1-A.**  *Under a subscription model, customers tend to perceive utilitarian goods more as hedonic than utilitarian ones.*

Additionally, according to previous literature, unlike other online shopping activities, SOS business is less exploratory and provides a new experience for consumers [33]. That is, consumers embrace the subscription model as a new shopping activity and experience curiosity and pleasure in the process [35]. This suggests that, if an existing product is provided via a different supply method such as a subscription model, this product will more likely be recognized as an experience good, even if the consumer knows it as a search good. Therefore, the quality inferences of consumers are predicted to shift from search to experience goods under a subscription model.

**H1-B.**  *Under a subscription model, customers tend to perceive search goods more as experience than search ones.*

Finally, as mentioned above, if a subscription model strengthens the hedonistic properties of a product, it may lower consumers’ intention to purchase. This is because hedonic goods are generally considered more luxurious and wasteful than utilitarian goods [65], instilling a sense of guilt in consumers when purchasing hedonic goods [66,67]. This feeling of guilt makes it difficult for consumers to justify their spending on hedonic goods [21]. Furthermore, according to the hedonic treadmill theory [68], the extent of happiness increased by consuming products and services diminishes over time [69]. For these reasons, consumers may become more cautious and selective in having a long-term, regular engaging relationship with suppliers. As such, if a subscription model is introduced, even in case of an existing product, consumers’ purchase intention will likely decrease.

**H1-C.**  *Under a subscription model, consumers’ purchase intentions tend to move from “yes” toward “no”.*

Previous research has suggested that utilitarian and hedonic shopping motives should be considered together in a study investigating consumer shopping behavior [70]. Consumption motivation is a fundamental driving force for customers’ purchase behavior and is hence an important and relevant component of retail marketing [19,71]. Additionally, quality inference (search versus experience goods) has also been discussed in several studies as an important factor that influences consumer purchase intention [24,55,72,73,74]. However, in the subscription economy model, it is difficult to find any previous studies that investigated both consumption motives and quality inferences to investigate factors affecting purchase intention. Which variable—consumption motivation or quality inference—has a more significant effect on subscription purchase intention? (RQ 2).

According to the literature on subscription models, utilitarian and hedonistic variables can influence customers’ purchase intentions [30,33,34,35]. Previous studies have shown that utilitarian goods stimulate repurchase intentions more strongly than do hedonic goods [34]. Bray et al. [15]’s findings suggest that consumers’ utilitarian motives have a greater impact on the decision to use subscription services than do their hedonic motives. Thus, consumption motivation affects the purchase intention of a product offered under a subscription model, and the purchase intention will likely increase when the customer perceives the product as a utilitarian vs. hedonic good.

**H2-A.**  *In a subscription model, when the consumption motivation defines a product as a utilitarian good, the purchase intention increases.*

Regarding quality inference, consumers were found to have higher purchase intention toward search goods than experience goods when purchasing online [24]. In the absence of any useful channel to access product information, the distinction between search and experience goods becomes more relevant given the low accessibility to experience goods [57]. However, other researchers argue that the distinction between search and experience goods has become blurred due to the spread of the Internet. As Klein [55] pointed out, the Internet allows consumers to obtain adequate product information on experience goods prior to purchase, which is traditionally congruent with search goods, thus blending the attributes of search and experience goods, as digital and network technologies reduce the information asymmetry that existed in offline settings in the past [75]. Thus, some scholars argue that providers should utilize differentiated strategies in selecting delivery channels and methods based on quality inference, while others assert that the advent of the Internet has blurred the distinction between search and experience goods. Will consumers perceive a product provided under a subscription model as an experience good, or will they recognize it as a search good? Does this distinction make sense in a subscription model? It is necessary to determine which of these perspectives regarding quality inference should be applied to the subscription model. Based on previous studies, this study hypothesizes that quality inference affects purchase intention, and the more a good is perceived as a search good, the higher the purchase intention becomes.

**H2-B.**  *In the subscription model, when the q* *uality inference defines a product as a search good, the purchase intention increases.*

Additionally, previous research has shown that consumer make decisions differently depending on the product type [21,22] and products may have deterministic attributes that make them more appealing to consumers [76]. Given their diverse nature, consumer attitudes and behaviors will likely vary depending on the type of subscription service being considered [15]. The category of subscription product type most suitable for the subscription economy can be predicted by deriving the sub-variable with the highest purchase intention among consumption motivation and quality inference, from Hypothesis 2 (H2-A and H2-B). If so, which product type categories generate the highest purchase intent under a subscription model? (RQ 3). 

This study aimed to empirically validate the predicted subscription product type categories. This will be done by further examining whether there are differences in the categories of subscription products that professionals and general consumers are more likely to purchase.

Experts are consumers who have a high understanding of product information and experience in using the products concerned and are highly familiar with them [58]. High product knowledge can influence consumer behavior [75,77]. Consumers with some level of product experience and knowledge have accumulated sufficient product information to enable them to process information [78,79]. Accordingly, the level of product knowledge has been treated as an important variable affecting information searches in the purchasing decision-making process [80,81]. Specifically, consumers with low product knowledge attempt to reduce their perceived risk of lack of knowledge by collecting product information [82] and use it in making purchase decisions, while consumers with high product knowledge make purchase decisions using existing information about products [83]. Therefore, the more knowledge and experience consumers have, the more selectively they obtain information in the information collection process [84] and the higher their self-confidence becomes, as they perform more empirical information processing. There is evidence that consumers’ knowledge level affects purchase intention and purchase decision [85], but there is little related research on the subscription model. If the product type categories preferred by consumer depending on the expertise, new insights will be provided for the providers who consider the subscription economy model. We hypothesize that experts and general consumers have different approaches to quality inference, and accordingly, they may have high purchase intention for different product type categories.

**H3.**  *Experts and consumers with different levels of knowledge of subscription models are more likely to purchase different categories of product types.*

## 3. Research Methodology 

### 3.1. Research Design and Procedure

The hypotheses formulated above were experimentally tested. A “one-group pretest–posttest design” was utilized for the 31 experts, and a “posttest-only control group design” for the 152 general consumers in South Korea. The one-group pretest–posttest design is a technique employed to ascertain the effect of experimental treatment by conducting pretest and posttest measurements for a single group [86]. It is primarily used by behavioral researchers to identify the effects of a treatment or intervention for a given sample [87]. The one-group pretest–posttest design is considered a better experimental design than one-off case studies in terms of measuring treatment effects. 

However, since factors other than treatment effects, i.e., confounders, can cause changes in the period between the pretest and posttest measurements, it is next to impossible to definitively prove causality [88]. Hence, given that exogenous variables are not strictly controlled, this design model is a much more viable option for exploring the possibility of effects than merely demonstrating a sophisticated causal relationship [86,88]. To overcome these problems, it was additionally examined whether the same results as the expert results can be obtained by the posttest-only control group design for general consumers. The posttest-only control group design has the advantage of preventing the pretest effect by not conducting the baseline measurements. 

**Figure 1:**
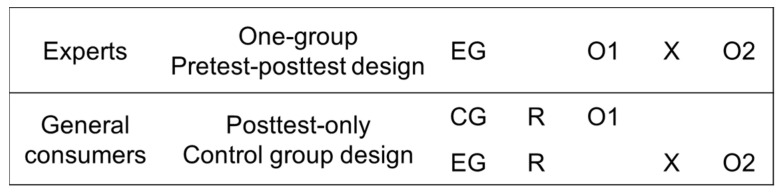
Experimental design.

Figure 1 presents the symbols for the research design. The acronyms represent the following: EG = experimental group subjected to treatment; CG = control group; X (exposure) = experimental stimulus; O (observation) = test; and R = randomization.

First, we administered an online survey to a group of 31 experts using a one-group pretest–posttest design. These experts had extensive knowledge of the subscription economy and business models as well as considerable careers or expertise in the relevant fields. To select individuals with the relevant practical and theoretical knowledge who were highly interested in new business models and trends, three pilot tests regarding the subscription economy and subscription models were conducted with members of famous business forum communities (HBR Forum Korea). This measure was undertaken to minimize response insincerity. Although a purposive sampling technique may not accurately represent the entire target population, it is a robust and reasonable cost method to collect more reliable data and accurate information and is thus widely used as a preliminary investigation method for problem discovery [89]. Purposive sampling is more efficient than convenience sampling when objective information about an ideal population is known in advance. Moreover, purposive sampling lends itself well to (1) small-scale expert investigations and (2) preliminary survey for basic or primary investigations before performing the main survey [90]. All 31 expert participants responded to both the pretest and posttest surveys, and they were given gift certificates worth approximately US $9 as a token of appreciation for participation. Given that the one-group pretest–posttest design involved administering surveys repeatedly to the same individuals, various measures were taken to ensure causality, such as minimizing the influence of the pretest and identifying and controlling potential interference events in the period between the pre- and post-measurements. In this study, the participants were not informed of the second survey when the first survey was being administered lest they should remember the survey items, which would undermine response sincerity and survey reliability due to a learning effect [91,92]. Furthermore, to minimize the learning effect associated with repeated administration of the survey, the first and second surveys were spaced two weeks apart. According to previous studies, respondents have difficulties with short-term memory and learning for longer than a day unless they have personal interest and an opportunity to review [93,94,95,96], and two to four weeks is an appropriate time interval for reducing a learning effect between two tests [97]. Accordingly, we estimated that a two-week interval between the two surveys would partially control errors caused by respondents remembering and learning from the first survey. 

Next, we also utilized a posttest-only control group design with 152 general consumers to determine whether identical results would be obtained. To this end, based on the Industrial Bank of Korea database, a survey was conducted with customers in their 20s or older who had the power to spend and who expressed their intention to participate. They were divided into four groups (two experimental groups and two control groups) and randomly assigned to a “pre” (pre-exposure to the subscription model) or “post” (post-exposure to the subscription model) group. The 99 subscription business cases were divided into two product groups: Product Group 1 (Cases 1 to 49) and Product Group 2 (Cases 50 to 99). This measure minimized the burden of the survey because, unlike experts, general consumers may have relatively low interest in the subscription model or may not have the motivation to respond to the survey for a long period of time. All subjects provided their informed consent for inclusion before they participated in the study. Table 1 summarizes the research design. 

### 3.2. Stimulus

For this study, 99 typical subscription business cases (the details of the 99 cases are provided in Appendix A) were collected. The 99 subscription business cases were collected by the researcher team from academic journals, media articles published after 2017, research publications from reputable institutions [93,98], and related books [28,99,100]. The actual business models and related details in these cases were checked and verified by visiting their official websites. 

Survey items were described in two different contexts: before and after the introduction of the subscription model (Table 2 and Table 3, respectively). The products, subscription fees, and subscription service contents presented in Table 3 were borrowed from actual subscription model companies. The questions regarding the product attributes were: “Do you perceive this product more as a utilitarian good or a hedonic good?” and “Do you perceive this product more as a search good or an experience good?” [101]. Purchase intentions were measured with the question, “Would you like to buy XXXX?” [102]. 

Each item was rated on a six-point semantic difference scale; utilitarian/hedonic, pertaining to consumption motive, and search/experience, pertaining to qualitative reasoning, were provided at each end of the scale.

This technique is useful for examining the differences, distance, and similarities between products and has been employed for various purposes, such as identifying respondents’ attitudes and characteristics, comparative product research, and company reputation surveys [103], across various fields of social sciences. The choice of an even- or odd-numbered scale depends on the number of neutral respondents. An odd-numbered scale is more suitable for studies that seek to predict the presence of respondents with a neutral attitude who must be identified for research purposes. In contrast, an even-numbered scale is more suitable when no neutral respondents are expected, or if the researcher wishes to prevent meaningless or evasive neutrality and identify its directionality, weak as it may be [90,103,104]. This concept is also referred to as a “forced rating scale”. This study utilized a six-point semantic differential scale to force the respondents to show their directional degree of inclination toward a particular type for all cases.

### 3.3. Measure

The electronic survey form was distributed to potential respondents. In the survey form, the purpose and aim of the study along with a cover letter contained the concepts of the study. Additionally, the legal acknowledgement of private data protection amendment statement was attached. All subjects received information regarding the purpose and use of response data before they participated in the study. Respondents confirmed their consent when they participated in the survey. The study was conducted in accordance with the Korean national law—Article 33 of the Statistics Act (Protection of Private Information) and the protocol fully observed national legislation law by providing designated information to the participant.

First, to determine whether consumers’ perceptions of product types and their purchase intentions change with the introduction of a subscription model for an existing product (H1-A, H1-B, H1-C), the results of the one-group pretest–posttest survey of the 31 experts were examined using a paired *t*-test. We also used an independent two-sample *t*-test to observe the same change in the perceptions of 152 general consumers.

Next, among the consumption motives and quality inferences, factors that influenced purchase intention after the introduction of the subscription model were identified (H2-A, H2-B). For this purpose, a multiple regression analysis was conducted using the consumption motivation/quality inference data of the expert group as an independent variable and purchase intention as a dependent variable. Additionally, we performed a hierarchical multiple regression analysis to confirm whether the statistical results obtained from the expert group are identical to those obtained from the general consumer group. 

Hierarchical multiple regression is typically used to assume that multiple independent variables influence the dependent variable and determine which independent variable produces the greatest effect.

**Figure 2:**
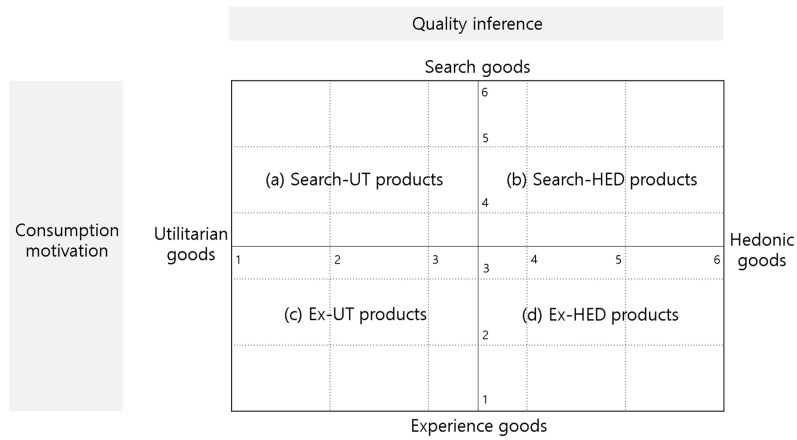
Classification of product type categories based on consumption motivation and quality inference.

Finally, product type categories with the highest consumer purchase intention were identified (H3). For this purpose, 4 product type categories were classified in a 2 × 2 combination based on the average values of consumers’ perceived consumption motives and quality inferences (Figure 2). 

Based on the median value of the six-point scale, consumption motives below the median value were classified as utilitarian goods and above the median value as hedonic goods. Similarly, quality inferences below the median value were classified as experience goods and above the median value as search goods (Codes for the four product type categories: search-utilitarian = Search-UT, search-hedonic = Search-HED, experience-utilitarian = Ex-UT, experience-hedonic = Ex-HED). 

Subsequently, one-way ANOVA was conducted using the four product type categories as independent variables and purchase intention as the dependent variable. One-way ANOVA tests whether there is a significant difference in the mean of the dependent variable in terms of an independent variable that comprises three or more groups. For the post-hoc analysis, we utilized the Scheffe test, which is used when there is a mean difference between groups due to a significant analysis result and can even be used with an unequal number of samples between groups [105]. For statistical analysis, SPSS 24.0 was used. 

### 3.4. Participants

Table 4 presents information regarding the 31 experts with practical knowledge and experience of the subscription economy. 

Table 5 presents the demographic profile of the 31 experts in the one-group pretest–posttest design, and Table 6 presents the demographic characteristics of the 152 general consumers subjected to the posttest-only control group design.

## 4. Results

### 4.1. Research Question 1: If a Subscription Model Is Introduced for an Existing Product, Will Consumers’ Perceptions of the Product’s Type and Their Purchase Intentions Change? (H1-A, H1-B, H1-C)

The results of the one-group pretest–posttest survey of the 31 experts were examined using a paired *t*-test. The changes in mean value, as shown in Table 7, indicate that significant differences occurred in terms of both product types and purchase intentions.

The mean value of consumption motivation was greater after the introduction of the subscription model (second survey) than before (first survey). This signifies that the response results of the second survey significantly shifted to the right (*t* = −10.238, *p* = 0.000). It means that the utilitarian goods perceived by the consumers may be recognized as the hedonic goods when the subscription model is applied. The mean value of the second survey was lower than that of the first for both quality inference and purchase intention. Specifically, quality inference shifted from search goods to experience goods (right to left, *t* = 3.037, *p* = 0.002), and purchase intention shifted from yes to no (right to left, *t* = 22.805, *p* = 0.000). 

Next, we observed whether the same changes occurred in the perceptions of 152 general consumers. Table 8 shows the results of the posttest-only control group design using an independent two-sample *t*-test.

Similar to the one-group pretest–posttest design, the consumption motives of the posttest-only control group shifted from utilitarian to hedonic ones (*t* = −11.276, *p* = 0.000). It was confirmed that when the subscription model is applied, the utilitarian goods perceived by the consumers may be recognized as the hedonic goods. Quality inference shifted from search goods to experience goods (*t* = 3.627, *p* = 0.000); purchase intentions shifted from yes to no (*t* = 26.046, *p* = 0.000). In both the one-group pretest–posttest design for the 31 experts and the posttest-only control group design for the 152 general consumers, the introduction of a subscription model led to significant changes in consumer perceptions of product types as well as purchase intentions. Therefore, all three hypotheses (H1-A, H1-B, H1-C) based on Research Question 1 were supported. 

**Figure 3:**
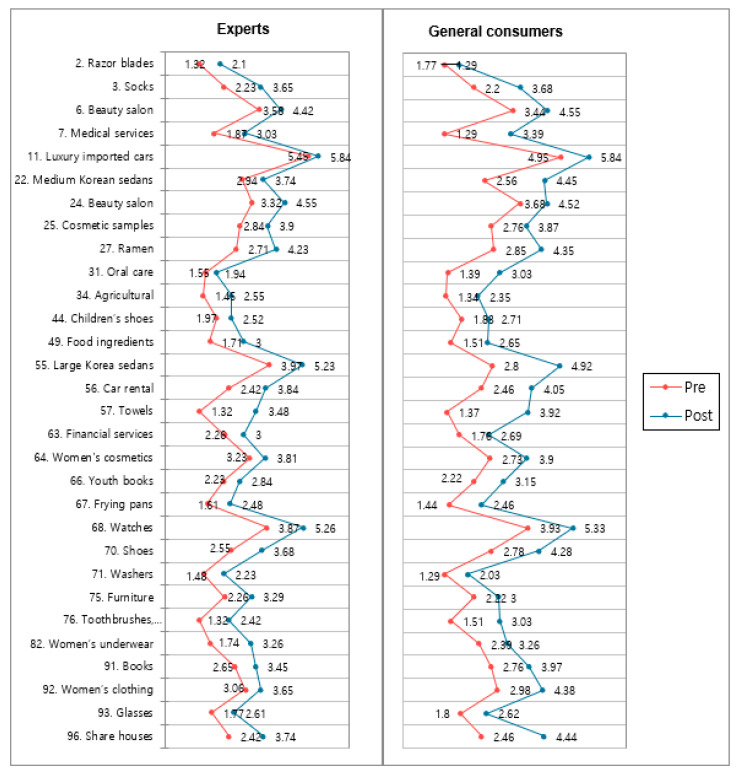
Items with significant *t*-test results in both experimental designs: consumption motivation.

**Figure 4:**
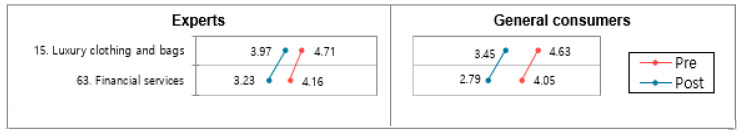
Items with significant *t*-test results in both experimental designs: quality inference.

**Figure 5:**
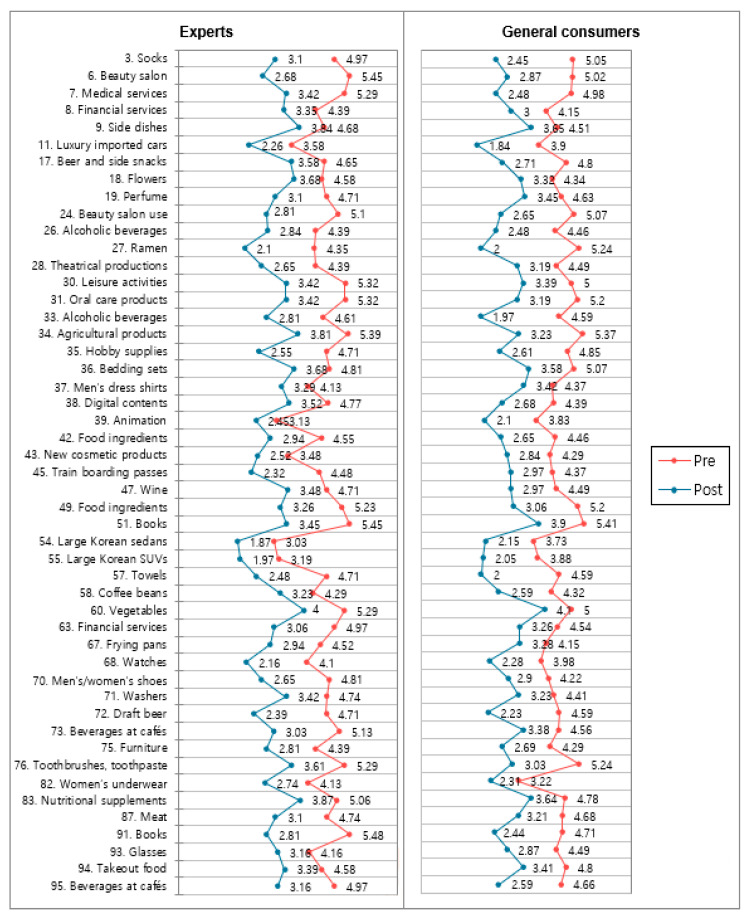
Items with significant *t*-test results in both experimental designs: purchase intention.

Figure 3, Figure 4 and Figure 5 present the lists of items among the 99 products that exhibited statistically significant results in both the one-group pretest–posttest design and the posttest-only control group design. In a total of 30 items, there was a significant result wherein consumption motives shifted from utilitarian to hedonic ones (*p* < 0.05). Two items showed significant shifts from search goods to experience goods (*p* < 0.05). Finally, a total of 49 items showed results wherein the purchase intention shifted from yes more to no. Products that are not present in these lists either only had one test showing statistically significant results or had both tests showing consistent but statistically insignificant directionality.

### 4.2. Research Question 2: Which Variable, Consumption Motivation or Quality Inference, Has a More Significant Effect on Subscription Purchase Intention? (H2-A, H2-B) 

First, a correlation analysis was conducted to determine the relationships between the variables. Table 9 outlines the analysis results. Consumption motivation and quality inference, the independent variables, were found to have a significant correlation with the dependent variable, purchase intention. In both the expert group and the general consumer group, quality inference showed a negative (–) correlation with purchase intention, and consumption motive showed a positive (+) correlation with purchase intention.

Next, a multiple regression analysis was conducted using the consumption motivation/quality inference data of the expert group as an independent variable and purchase intention as a dependent variable (Table 10).

The regression equation significantly predicted purchase intention (F = 33.662, *p* = 0.000), whereby consumption motivation was identified as a significant predictor of purchase intention in the subscription economy. Although the *R*^2^ value is not high, it does not mean that it is not worth interpreting or is useless [106]. In the subscription economy, better explanatory power is obtained if the model includes all predictors that influence purchase intention. However, the point of the model is not to validate unambiguous predictors, but to guess and identify small but definitely related variables. In such studies, even small effect sizes can have scientific or clinical significance [106]. The problem of multicollinearity, in which indicates high correlations among independent variables, can be checked using the variance inflation factor (VIF) value. With the VIF value not exceeding 10, it was demonstrated that there was no problem of multicollinearity. After confirming the significance of the main variables, consumption motivation was found to have a negative effect on purchase intention. In other words, a lower consumption motivation score indicates a higher purchase intention, which suggests that purchase intention increases when a consumer perceives a good as being more utilitarian than hedonic based on the scale used in this study. In contrast, quality inference did not show any significant effect on purchase intention (*p* = 0.194). 

Additionally, we performed a hierarchical multiple regression analysis to confirm whether the statistical results obtained from the expert group are identical to those obtained from the general consumer group (Table 11). 

Since Model 1 (F = 154.950), Model 2 (F = 77.774), and Model 3 (F = 51.835) all showed significant values, the regression line can be considered appropriate for the models. All tolerance limits were between 0.1 and 10, indicating that there was no problem of multicollinearity. 

In the first process step of hierarchical multiple regression, consumption motivation was added in Model 1 as the input variable, which revealed a statistically significant influence on purchase intention (*β* = −0.207, *p* = 0.000). In the second step, quality inference was added in Model 2 as the input variable. Since Model 2 has a slightly lower fit (adjusted *R*^2^ = 0.042, F = 77.744) than Model 1, the quality inference added to Model 2 had no significant influence on the model’s overall explanatory power and the dependent variable, purchase intention (*β* = −0.013, *p* = 0.433). In the final step, group classification within general consumers (Group 1: 1 to 49, Group 2: 50 to 99) was converted into a dummy variable to examine whether group classification had an effect on purchase intention. Model 3 did not show any significant change in fit, and the group classification within general consumers did not significantly affect purchase intention (*β* = 0.001, *p* = 0.954).

These findings indicate that, under the subscription model, only consumption motivation had a statistically significant effect on the purchase intention of both experts and general consumers. Specifically, the more a product in a subscription model is perceived as utilitarian vs hedonic, the greater the purchase intention. Thus, H2-A was supported. However, quality inference did not have a statistically significant influence on purchase intentions for both the experts and the general consumers. Therefore, H2-B was rejected. 

### 4.3. Research Question 3: Which Product Type Category Generates the Highest Purchase Intention under the Subscription Model? (H3)

Finally, we empirically analyzed the relationship between product type categories classified according to consumers’ perceptions and purchase intentions. Table 12 shows the 99 products grouped into four product type categories. The number of products falling under each category changed according to the change in the perception of the product by both experts and general consumers.

Table 13 presents the test results regarding differences in purchase intentions and product type categories as perceived by the 31 experts before and after the subscription model was introduced. The one-way ANOVA results demonstrated significant differences both before (F = 23.815, *p* = 0.000) and after (F = 9.428, *p* = 0.000) the introduction of the subscription model, and among the product type categories, Ex-UT products generated higher purchase intention than the other product type categories. Thus, the purchase intentions of the 31 experts were found to be the highest for Ex-UT products both before and after the subscription model was introduced.

To determine whether the same results were obtained in the group of 152 general consumers, one-way ANOVA was conducted based on the posttest-only control group design data. Table 14 presents the test results regarding differences in purchase intentions and product type categories as perceived by the different groups before and after the introduction of a subscription model. The one-way ANOVA results demonstrated significant differences both before (F = 12.245, *p* = 0.000) and after (F = 16.972, *p* = 0.000) the subscription model was introduced. As was the case with the expert group, purchase intentions for Ex-UT products were higher than those for the other products before introducing the subscription model in the control group. However, after introducing the subscription model, Search-UT products generated higher purchase intentions than the other product type categories. 

Hypothesis 3 was supported by the difference in the product type category that generates high purchase intentions for experts and general consumers in the subscription model.

## 5. Discussion and Conclusions

The main goal of the article was to examine changes in consumer perceptions of product types and purchase intentions when a subscription model was introduced for products normally sold on a one-time basis. Additionally, based on the changed consumer perceptions, this study sought to determine the product type categories better-suited for the subscription model and to identify the main variables that affect purchase intention. As an initial exploratory attempt on this topic, the current study reviewed 99 subscription businesses through literature reviews and media articles. We examined 99 existing subscription business cases under two contexts—namely, before and after the introduction of the subscription model by administering surveys for each context. The survey respondents included 31 experts with extensive knowledge of the subscription economy and 152 general consumers in South Korea. We performed a reverification procedure to determine whether similar results were obtained for both groups. Paired *t*-test, independent sample *t*-test, multiple regression analysis, hierarchical regression analysis, and ANOVA were used as analysis methods.

First, as a result of the *t*-test, after the subscription model was introduced for an existing product, consumption motivation shifted from utilitarian to hedonic motives, quality inference shifted from search to experience attributes, and purchase intentions shifted from “yes” to “no”. Therefore, Hypotheses H1-A, B, C were adopted. Studies of behavioral decision-making suggest that consumers tend to make decisions based on context rather than previously established rules of choice when placed in a particular situation [25,26]. The context effect explains that consumers create new preferences according to the context of a given situation [42,43,63]. We speculated that the subscription model could have a context effect strong enough to stimulate customer awareness into converting customer relationships with existing products and services into lasting relationships rather than one-time sales. The fact that the introduction of a subscription model can change the customer perception of existing products can serve as an important basis for expanding research on subscription models in the future.

Second, this study examined the effects of consumption motivation and quality inference on purchase intention under a subscription model through regression analysis. The results showed that consumption motivation affected purchase intention, in experts and general consumers alike, while quality inference did not exhibit statistically significant results. Specifically, the greater the consumer perception of a product as a utilitarian rather than hedonic one, the higher the purchase intention under a subscription model. Thus, H2-A was adopted but H2-B was rejected. These results support the findings of previous studies that utilitarian goods are a better predictor of repurchase intentions than hedonic goods [34] and that the utilitarian motivations of consumers have a greater influence on subscription service use than do hedonic motivations [15]. In contrast, quality inference did not show statistically significant results regarding subscription purchase intentions. These results support those of a number of studies that indicate that the distinction between experience goods and search goods has become blurred due to the spread of the Internet [61,107,108,109].

Finally, as a result of the ANOVA analysis, prior to introducing the subscription model, both experts and general consumers showed the highest purchase intentions toward Ex-UT products. However, after the subscription model was introduced, search-UT products generated the highest purchase intentions among the general consumers, while Ex-UT products still generated the highest purchase intentions in the expert survey. The differences in the results regarding the product type categories that generated high purchase intentions after the subscription model was introduced can be attributed to the respondents’ different levels of understanding and interest in subscription models. In other words, the expert subjects either worked in industries related to the subscription economy or had relevant theoretical knowledge, which explains why they preferred Ex-UT products whose quality could be evaluated through direct observation or use under a subscription model. In contrast, the general consumers, who are potentially more conservative, preferred Search-UT products whose quality could be predicted before use via external information. These results are in line with the existing research finding that consumers’ knowledge level affects their purchasing decisions also under a subscription model [85]. Furthermore, these results are consistent with those of existing studies that consumers who lack accumulated information compared to experts are more likely to make decisions based on external peripheral properties and reduce perceived risk by collecting product-related information [79,82,110]. Therefore, H3 was adopted. This finding has an important practical implication for providers by suggesting product type categories that are suitable for their subscription models. 

Thus, according to this study’s findings relating to the changes in consumer perception, products with utilitarian and search attributes are more appropriate for a company that chooses a long-term, recurring subscription model over a one-time purchasing model (RQ 1). However, while consumers’ purchase intentions increased when they perceived a good as utilitarian, quality inference did not have a statistically significant effect on their purchase intentions (RQ 2). Therefore, a business using a subscription model should primarily focus on products with strong utilitarian attributes to ensure initial success. Furthermore, while experts with extensive knowledge of the subscription model demonstrated a high purchase intention toward Ex-UT products, the more conservative general consumers demonstrated a higher purchase intention toward Search-UT products (RQ 3). Hence, subscription-model businesses must undertake product selection and targeting strategies after determining the product type preferences of their target consumer group.

## 6. Implications, Limitation and Future Research Direction

### 6.1. Theoretical Contributions

The findings of this study have the following theoretical implications. Previous studies were limited in terms of geographical focus (most studies set in the US) and an emphasis on specific types of subscriptions [15]. This study investigated consumers’ perceptions of 99 real companies to which subscription models were introduced, not limited to specific industries. The results of this study, which explored changes in consumers’ perceptions caused by introducing a subscription model, provide empirical data useful for future research. Specifically, this study provides new insights into the effect of introducing the subscription model by verifying that changes in consumer perceptions of product attributes and purchase intentions can occur due to context effects. In the past two decades, few studies regarding the context effect have been conducted from an online perspective [111]. 

In addition, the existing research was expanded by applying and verifying consumption motive (utilitarian goods versus hedonic goods) and quality inference (experience versus search goods) variables, which were discussed as major influencing factors on one-time purchase intentions, to the subscription economy model.

### 6.2. Practical Implications

This study contributes in confirming that introducing a subscription model may change consumers’ perceptions and identifying the product types that suppliers should prioritize when initially introducing a subscription model. From a practical perspective, businesses must realize that a subscription model is not necessarily created by merely adding regularity to the delivery of existing products and should strive to understand consumers’ perceptions regarding their products and adopt suitable strategies when introducing a subscription model. The core of the subscription economy is consumer-oriented thinking. Businesses must consistently offer relational values adjusted to consumers’ needs and preferences to retain their willingness to pay. As such, communication and sales approaches should be based on differentiated marketing strategies based on the consumers’ perceptions of the types of products the subscription model offers. 

In addition, this research was conducted with the experts and consumer group in Republic of Korea (South Korea). According to report issued by one of the prominent research institution—KT (Korea Telecom) Economy & Management Research Institution, the size of South Korean subscription economy increased by 54.8% from 25.9 trillion KRW in 2016 to 40.1 trillion in 2020. It is also forecasted the market size will grow up to 100 trillion Korea in 2025. Especially in the post-COVID-19 stage, South Korea’s well-established IT infrastructure enhances non face-to-face purchase patterns among the general consumption group. Accordingly, it is expected to provide insights from South Korea to countries with similar market environments or growth patterns of the subscription economy.

### 6.3. Limitations and Future Research Directions

Since this study involved an exploratory investigation of changes in consumer perception and purchase intention, it could not identify specific factors that led to changes in consumer perception. In addition, although the ANOVA algorithm identified product type categories suitable for the subscription economy, it did not specifically account for the engagement factors or barriers that attract consumers to subscription offers. 

Although this study deals with the search for consumption motivation and quality inference as factors influencing purchase intention in the subscription economy, it is necessary to explore various factors in the future. Purchase intention is an important variable of consumer behavior, which has been influenced by many consumers’ internal and external factors. However, in this study, two selected variables, consumption motive and quality inference, were considered to explain this complex phenomenon. In addition, only consumption motivation was significant and quality inference was not. 

To address this limitation, further research is needed to investigate the main causes of changes in consumer perception under a subscription model. Further research is also needed to investigate specific changes in each industry to identify which product groups are most impacted by the introduction of the subscription model.

Finally, this study showed the differences between groups in the demographic profile of respondents. In the future, a chi-square test may be performed to investigate the resulting differences. Others can also think about how the differences might affect the results of their studies.

## Figures and Tables

**Table 1:** Research design.

Survey	Expert (*n* = 31)	General Consumer (*n* = 152)	
Pre	(One group) Nos. 1–99	(Group 1) Nos. 1–49	(Group 2) Nos. 50–99
Post		(Group 3) Nos. 1–49	(Group 4) Nos. 50–99

**Table 2:** Example of survey items (before introduction of subscription model).

Case 1. Contact Lens			
Consumption Motivation	Utilitarian goods	①—②—③—④—⑤—⑥	Hedonic goods
Quality Inference	Experience goods	①—②—③—④—⑤—⑥	Search goods
Purchase Intention	No	①—②—③—④—⑤—⑥	Yes

**Table 3:** Example of survey items (after introduction of the subscription model).

Case 1. Hubble—Contact Lens Subscription Monthly Delivery of Customer-Selected Contact Lenses for $28 Per Month			
Consumption Motivation	Utilitarian goods	①—②—③—④—⑤—⑥	Hedonic goods
Quality Inference	Experience goods	①—②—③—④—⑤—⑥	Search goods
Purchase Intention	No	①—②—③—④—⑤—⑥	Yes

**Table 4:** Subjects of the one-group pretest–posttest design (31 experts).

1	18 years of experience as the head of digital marketing
2	18 years of experience in digital marketing and as a freelance English interpreter and translator
3	18 years as a reporter; head of new business/innovation
4	8 years on the future business planning team of a Korean steel company, P
5	Meta-branding strategic planning executive, Company L Economic Research Institute
6	Head of in-house venture company A, the largest beauty company in Korea; CSO of startup
7	Founder and CEO of steel recycling startup
8	10 years in the futures business of a financial holding company, N
9	10 years as the project manager (PM) of a global energy company and heavy industry marketing
10	20 years in digital-development system integration (SI) for an IT service company, L
11	10 years as a marketer in the beauty industry; reading community partner
12	Head of new business at a communication company, S; marketer
13	Power blogger; marketer at large Korean distributor company, E
14	New business in the financial sector; head of planning at the Korea Undergraduate Association of STEM
15	Founder of a health social venture; startup marketer
16	PR manager at an e-commerce & InsurTech company
17	20 years as the team leader of a platform business team; financial SI expert; MBA
18	10 years in financial SI planning; section manager of platform business team
19	Market designer, Growth Lab team leader at Company T, a foreign language learning subscription startup
20	Startup business legal advisor; completed Korean Bar Association Startup Academy
21	Founder of software education startup company A, recognized as technologically innovative startup and accelerator
22	3 years as the manager of new business and big data at a large Korean distribution company, L
23	COO of a startup; startup and IT headhunter; Silicon Valley Connector
24	University student, social venture founder, and writer
25	15 years of experience in advertising and planning, and as a digital marketer and brand manager
26	10 years of experience as an online and offline marketer and in financial big data planning
27	15 years of experience in financial enterprise digital and fintech planning
28	15 years as a financial SI; PM of 30 projects
29	Doctor of Business Administration, fields: technology management, product and service innovation
30	Startup accelerator: 25 years as the CEO of a startup
31	Head of raw material importing and purchasing team at a large Korean food company; international trade history

**Table 5:** Experts: Demographic profile of respondents in the one-group pretest–posttest design.

Category		Frequency	Ratio
Gender	Male	17	54.8
	Female	14	45.2
Age	20s	6	19.4
	30s	12	38.7
	40s	12	38.7
	50s	1	3.2
	60s or older	0	0.0
Marital status	Single	19	61.3
	Married	12	38.7
Education	High school graduate	-	-
	Attending university	2	6.4
	University graduate	14	45.2
	Attending graduate school	3	9.7
	Graduate school graduate	12	38.7
Monthly expenditures (1 USD = 1163.5 KRW, 22 September 2020)	Less than $858	3	9.7
	$858–$2575	15	48.4
	$2575–$4293	10	32.2
	$4.293–$6010	3	9.7
	$6010 or more	-	-

**Table 6:** General consumers: demographic profile of respondents in the posttest-only control group design.

Measurement Item		Pre		Post	
		1–49	50–99	1–49	50–99
Group classification		Group 1	Group 2	Group 3	Group 4
N (=152)		41	41	31	39
Gender	Man	12 (29.3)	30 (73.2)	15 (48.4)	19 (48.7)
	Woman	29 (70.7)	11 (26.8)	16 (51.6)	20 (51.3)
Age	20s	1 (2.4)	7 (17.1)	14 (45.2)	6 (15.4)
	30s	12 (29.3)	22 (53.7)	14 (45.2)	21 (53.8)
	40s	27 (65.9)	11 (26.8)	3 (9.6)	10 (25.6)
	50s	1 (2.4)	1 (2.4)	-	1 (2.6)
	60s or older	-	-	-	1 (2.6)
Marital status	Single	8 (19.5)	19 (46.3)	23 (74.2)	14 (35.9)
	Married	33 (80.5)	22 (53.7)	8 (25.8)	25 (64.1)
Education	High school graduate	1 (2.4)	1 (2.4)	-	3 (7.7)
	Attending university	-	4 (9.8)	8 (25.8)	5 (12.8)
	University graduate	33 (80.5)	26 (63.4)	18 (58.1)	29 (74.3)
	Attending graduate school	3 (7.3)	1 (2.4)	1 (3.2)	1 (2.6)
	Graduate school graduate	4 (9.8)	9 (22.0)	4 (12.9)	1 (2.6)
Monthly expenditures (1 USD = 1163.5 KRW, 9.22.20)	Less than $858	3 (7.3)	8 (19.5)	11 (35.5)	9 (23.1)
	$858–$2575	16 (39.0)	20 (48.8)	15 (48.4)	11 (28.2)
	$2575–$4293	13 (31.8)	6 (14.6)	5 (16.1)	13 (33.3)
	$4293–$6010	6 (14.6)	4 (9.8)	-	4 (10.3)
	$6010 or more	3 (7.3)	3 (7.3)	-	2 (5.1)

**Table 7:** Experts: paired *t*-test results for one-group pretest–posttest design.

	Variable	N	Response Difference (Pre–Post)		*t*	*p*
			Mean	SD		
Response 1	Consumption motivation	3069	−0.306	1.656	−10.238	0.000
Response 2	Quality inference	3069	0.117	2.140	3.037	0.002
Response 3	Purchase intention	3069	0.910	2.210	22.805	0.000

**Table 8:** General consumers: independent two-sample *t*-test results for posttest-only control group design.

Variable	Category	Survey Questions					
		Total (1–99)		Product Group 1 (1–49)		Product Group 2 (50–99)	
		Pre	Post	Pre	Post	Pre	Post
Consumption Motivation	N	4059	3469	2009	1519	2050	1950
	Mean	3.29	3.79	3.33	3.77	3.25	3.80
	SD	1.954	1.850	2.008	1.907	1.900	1.805
	DOF	7526		3526		3998	
	*t*	−11.276		−6.559		−9.413	
	*p*	0.000		0.000		0.000	
Quality Inference	N	4059	3469	2009	1519	2050	1950
	Mean	3.19	3.04	3.21	3.04	3.23	3.10
	SD	1.789	1.770	1.812	1.775	1.785	1.779
	DOF	7526		3.526		3998	
	*t*	3.627		2.668		2.233	
	*p*	0.000		0.008		0.026	
Purchase Intention	N	4059	3469	2009	1519	2050	1950
	Mean	3.98	2.91	4.15	2.91	3.81	2.91
	SD	1.852	1.687	1.860	1.690	1.829	1.686
	DOF	7526		3526		3998	
	*t*	26.046		20.386		16.247	
	*p*	0.000		0.000		0.000	

(N = number of samples, SD = Standard deviation, DOF = Degree of Freedom, *t* = *t*-test statistic, *p* = significance probability).

**Table 9:** Results of the correlation analysis between the main variables.

	Variables	Consumption Motivation	Quality Inference	Purchase Intention
Experts	Consumption motivation	1		
	Quality inference	0.055 (0.002)	1	
	Purchase intention	−0.212 (0.000)	0.058 (0.001)	1
General consumers	Consumption motivation	1		
	Quality inference	0.151 (0.000)	1	
	Purchase intention	−0.207 (0.000)	−0.044 (0.009)	1

**Table 10:** Experts: Results of multiple regression analysis to identify factors influencing purchase intention.

Dependent Variable	Independent Variables	B	*β*	*t*	*p*	VIF
Purchase intention	(Constant)	4.413				
	Consumption motivation	−0.147	−0.147	−8.178	0.000	1.006
	Quality inference	0.026	0.023	1.300	0.194	1.006
*R*^2^ = 0.021, Δ*R*^2^ = 0.021, F = 33.662 (*p* = 0.000)						

(B = estimates, *β* = Standardized estimates, *t* = *t*-test statistic, *p* = significance probability, VIF = Variance Inflation Factor).

**Table 11:** General consumers: results of hierarchical multiple regression analysis to identify factors influencing purchase intention.

Model	Independent Variables	B	*β*	*t*	*p*	VIF
1	(Constant)	3.622		56.737	0.000	
	Consumption motivation	−0.189	−0.207	−12.448	0.000	1.000
	*R*^2^ = 0.043, Δ*R*^2^ = 0.043, F = 154.950 (*p* = 0.000)					
2	(Constant)	3.654		48.524	0.000	
	Consumption motivation	−0.187	−0.205	−12.187	0.000	1.023
	Quality inference	−0.013	−0.013	−0.784	0.433	1.023
	*R*^2^ = 0.043, Δ*R*^2^ = 0.042, F = 77.774 (*p* = 0.000)					
3	(Constant)	3.652		44.735	0.000	
	Consumption motivation	−0.187	−0.205	−12.185	0.000	1.023
	Quality inference	−0.013	−0.013	−0.784	0.433	1.023
	Product group (1:nos 1–49/2:nos 50–99)	0.003	0.001	0.057	0.954	1.000
	*R^2^* = 0.043, Δ*R^2^* = 0.042, F = 51.835 (*p* = 0.000)					

(B = estimates, *β* = Standardized estimates, *t* = *t*-test statistic, *p* = significance probability, VIF = Variance Inflation Factor).

**Table 12:** Ninety-nine cases classified according to 4 product type categories.

Product Type Categories	Experts		General Consumers	
	Pre	Post	Pre	Post
a. Search-UT products	17	5	14	2
b. Search-HED products	15	18	20	12
c. Ex-UT products	38	39	44	41
d. Ex-HED products	29	37	21	44
Total	99	99	99	99

**Table 13:** One-way ANOVA results of purchase intention by product type category for the experts.

	Product Type Categories	N	M	SD	F (*p*)	Scheffe
Pre	(a) Search-UT products	527	4.15	1.810	23.815 (0.000)	c > a, d, b
	(b) Search-HED products	465	3.39	1.852		
	(c) Ex-UT products	1178	4.20	1.822		
	(d) Ex-HED products	899	3.92	1.851		
Post	(a) Search-UT products	155	3.18	1.793	9.428 (0.000)	c > a, b, d
	(b) Search-HED products	558	2.94	1.744		
	(c) Ex-UT products	1209	3.27	1.710		
	(d) Ex-HED products	1147	2.93	1.637		

(N = number of samples, M = Mean, SD = Standard deviation, F = F-Value, *p* = significance probability).

**Table 14:** One-way ANOVA results of purchase intention by product type category for the general consumers.

	Product Type Categories	N	M	SD	F (*p*)	Scheffe
Pre	(a) Search-UT products	574	3.65	1.801	12.245 (0.000)	c > d, b, a
	(b) Search-HED products	820	3.88	1.878		
	(c) Ex-UT products	1804	4.15	1.835		
	(d) Ex-HED products	861	3.94	1.861		
Post	(a) Search-UT products	70	3.44	1.682	16.972 (0.000)	a > c, d, b
	(b) Search-HED products	420	2.75	1.745		
	(c) Ex-UT products	1423	3.12	1.687		
	(d) Ex-HED products	1556	2.73	1.652		

(N = number of samples, M = Mean, SD = Standard deviation, F = F-Value, *p* = significance probability).

## Appendix A

**Table A1:** 99 Cases of Subscription Business.

No.	Company	Product	No.	Company	Product
1	Hubble	Contact lenses	2	Wisely	Razor blades
3	MEHISOX	Socks	4	Happy Moon Day	Organic sanitary napkins (Female hygiene)
5	Kindoh	Diapers	6	Monthly Hair	Services in beauty salon
7	Forward Healthcare	Medical services	8	Charles Schwab	Financial services
9	The Banchan	Side dishes	10	Laundrygo	Laundry service
11	Porsche Passport	Cars	12	Netflix	Media content
13	Millie’s Library	E-books	14	Pinzle	Art publishing
15	Closet Share	Luxury clothing	16	Hooch	Alcoholic beverages
17	Veluga Brewery	Alcoholic beverages and snacks	18	Kukka	Flowers
19	Paffem	Perfume	20	MS XBOX	Video games
21	Bark Box	Dog supplies	22	Hyundai Selection	Cars
23	Loot Crate	Game equipment	24	MEZON	Services in beauty salon
25	IPSY	Cosmetics	26	GUBIT	Alcoholic beverages
27	Yaro Ramen	Ramen	28	Kangeki	Theatrical productions
29	Fitty	Exercise	30	Leisure me	Leisur (Sports, tourism)
31	Quip	Oral care	32	LOLA	Tampons (Female hygiene)
33	Daily Shot	Alcoholic beverages	34	Murung Farm	Agricultural products
35	Hobby in the Box	Hobby supplies	36	Clean Bedding	Bedding
37	Weekly Shirts	Men’s dress shirts	38	PUBLY	Digital content (trend, career skill)
39	Laftel	Animation streaming	40	Open Gallery	Artwork
41	Dollar Shave Club	Razor blades	42	Blue Apron	Food ingredients
43	Birchbox	Cosmetics	44	Nike Adventure Club	Children’s shoes
45	SNCF	Train boarding passes	46	Inoshave	Shaving supplies
47	Purple Dog	Wine	48	Dolo Box	Pet supplies
49	Hello Fresh	Food ingredients	50	OFFICE PASS	Office supplies
51	Flybook	Books	52	Kyobo SAM	E-books
53	All the Time MINI	Cars	54	Hyundai Genesis Spectrum	Cars
55	KIA Flex	Cars	56	Socar Pairing	Cars
57	Noble Made	Towels	58	Fritz	Coffee
59	Sooldamhwa	Traditional liquor	60	Mannabox	Fresh food
61	Doctors Me	Regular specialist consultations	62	Deli Shirts	Men’s dress shirts
63	Wealth front	Financial services	64	Walmart	Cosmetics
65	Graze	Snacks	66	Owlcrate	Youth books
67	Lafeeolla	Cooking pan replacements	68	Karitoke	Watches
69	Peloton	Exercise	70	ShoeDazzle	Shoes
71	Bundle	Washing machines	72	Kirin Hometap	Draft beer
73	Wemakeprice W café	Coffee	74	SERENDIP	Book summaries
75	Feather	Furniture	76	Doctor Noah	Oral care
77	Flier	Book summaries	78	Bean Brothers	Coffee
79	Nescafe Capsule To door	Coffee capsules	80	SM LYSN	Fanclub service
81	YouTube Premium	Online video content	82	Adore Me	Underwear
83	Care of	Nutritional supplements	84	PORTO	Reference books
85	Toun28	Cosmetics	86	Snack Nation	Snacks for companies
87	Farmision	Meat	88	The Vegan Kind	Vegan products
89	Pact	Coffee	90	Glossy Box	Cosmetics
91	The Willoughby Book Club	Books	92	Air Closet	Clothing
93	NINAL	Glasses	94	POTLUCK	Food
95	Handel’s Café	Beverages	96	ADDress	Share house
97	Le Tote	Clothing	98	Zwift	Indoor cycling game
99	Unique Your Nail	Nail art			
